# A systematic review of the quality of studies on dementia prevalence in Italy

**DOI:** 10.1186/s12913-016-1741-7

**Published:** 2016-09-21

**Authors:** Gianluca Bruti, Elisabetta Cavallucci, Michele Mancini, Alessandro Bitossi, Marzia Baldereschi, Sandro Sorbi

**Affiliations:** 1 Eli Lilly Italia S.p.A., Via A Gramsci 731/733, 50019 Sesto Fiorentino, FI Italy; 2 Institute of Neurosciences, Italian National Research Council, Via Madonna del Piano 10, 50019 Sesto Fiorentino, FI Italy; 3 Department of Neuroscience, Psychology, Drug Research and Child Health, University of Florence, Viale Pieraccini 6 Firenze and Centro IRCCS “Don Carlo Gnocchi”, Via di Scandicci 269, Florence, Italy

**Keywords:** Alzheimer’s disease, Dementia, Epidemiology, Italy

## Abstract

**Background:**

Dementia, including Alzheimer’s disease (AD), is one of the most burdensome medical conditions. In order to better understand the epidemiology of dementia in Italy, we conducted a systematic search of studies published between 1980 and April 2014 investigating the prevalence of dementia and AD in Italy and then evaluated the quality of the selected studies.

**Methods:**

A systematic search was performed using PubMed/Medline and Embase to identify Italian population-based studies on the prevalence of dementia among people aged ≥60 years. The quality of the studies was scored according to Alzheimer’s Disease International (ADI) criteria.

**Results:**

Sixteen articles on the prevalence of dementia and AD in Italy were eligible and 75 % of them were published before the year 2000. Only one study was a national survey, whereas most of the studies were locally based (Northern Italy and Tuscany). Overall, the 16 studies were attributed a mean ADI quality score of 7.6 (median 7.75).

**Conclusions:**

Available studies on the prevalence of dementia and AD in Italy are generally old, of weak quality, and do not include all regions of Italy. The important limitations of the few eligible studies included in our analysis, mostly related to their heterogeneous design, make our systematic review difficult to interpret from an epidemiologic point of view. Full implementation of a Dementia National Plan is highly needed to better understand the epidemiology of the disease and monitor dementia patients.

## Background

### Rationale

Dementia, including Alzheimer’s disease (AD), is one of the most burdensome medical conditions. As the prevalence of dementia increases with age, the number of people living with this condition is expected to surge in the next few decades as people live longer.

What is known about the epidemiology of dementia in Italy comes from prevalence data which typically have been generated from studies that may be inconsistent regarding diagnostic criteria, age of the studied population, or assessments used [1–3]. While there are also many publications on the prevalence of dementia in Western European countries, the results of these studies vary considerably [4]. There is therefore an urgent need to come to a methodological consensus on how best to design epidemiological studies of dementia [2].

One important determinant of a reliable evaluation is the quality of the studies examined. In general, the quality of prevalence studies of dementia to date reflects the difficulty of diagnosing dementia. It has been suggested that a dementia diagnosis should be based upon a multidomain cognitive test battery, an informant interview, and a structured disability assessment, as well as a clinical interview to eliminate other causes of cognitive impairment [4]. Following very restricted criteria, Prince and colleagues [4] selected and ranked 51 European studies according to the standardized scoring system described in the 2009 Alzheimer Disease International (ADI) report [5] (Appendix). In this very comprehensive work, the European studies yielded a good (± standard deviation) mean quality score, with 8.2 ± 1.8 points (range of mean ± standard deviation quality scores of the studies across all regions: 5 ± 0.7 to 9.7 ± 2; overall mean all-region quality score: 7.9 ± 2) although only 8 % of the studies referred to post-2000 research. This suggests that data are lacking on the prevalence of dementia in Europe in the last decade [4]. Nevertheless, the authors confirmed the prevalence data reported by the European Collaboration for Dementia Group (EuroCoDe) [1], with an age and gender standardized prevalence of 7.3 %, which is very similar to the 7.1 % prevalence previously estimated by the EuroCoDe group [1].

Another important systematic review was conducted on prevalence data of dementia in Europe by the Alzheimer Cooperative Valuation in Europe (ALCOVE) [6]. This systematic review took into consideration both the quality of the studies (according to the quality score proposed by the 2009 ADI report [5]; Appendix) and the use of standardized clinical criteria (e.g. Diagnostic and Statistical Manual of Mental Disorders, DSM) [5, 7]. According to these methods, only 3 of the 17 studies selected in the EuroCoDe review [1] and 10 of the 12 studies in the ALCOVE review [6] adopted the clinical criteria of the DSM-IV. The DSM-IV criteria were chosen as a benchmark by the authors because it was the most frequently used method in these epidemiological studies. The mean quality score of all studies that adopted the DSM-IV criteria was 6.85 ± 1.93 (median 7, range 4.5–10.5), whilst in those with a quality score ≥7 the authors found a prevalence of dementia of 7.2 %. The use of these stringent criteria led to a mean decrease of 22 % in total rate for dementia, compared with the EuroCoDe review estimates [1], and to a mean decrease of 12 % in the total rate of dementia, compared with ALCOVE review estimates [6].

### Objectives

The objectives of this systematic review were: (i) to conduct a review of studies on prevalence of dementia in Italy; and (ii) to evaluate the quality of identified studies according to the standardized scoring system for the assessment of epidemiological trials in dementia.

## Methods

We estimated the quality of studies conducted on the prevalence of dementia and AD in Italy by carrying out a systematic review of the Italian literature published between January 1st 1980 and April 1st 2014, using PubMed/Medline and Embase and searching for the following terms (in any field): (dementia OR Alzheimer disease OR Alzheimer’s disease) AND prevalence AND Italy, with no language restriction. We sought and included all Italian population-based studies on the prevalence of dementia among people with age equal to or greater than 60 years old. In order to get a comprehensive ranking of the quality of studies published to date on the prevalence of dementia and AD in Italy, we excluded only the following papers:

1. Studies of prevalence from the follow-up phase of a population cohort;
2. Studies of nursing home or residential care populations, primary care attendees, or other unrepresentative service-user populations;
3. Studies in which the ascertainment of dementia depended upon help-seeking and/or receipt of dementia care services;
4. Studies restricted to young-onset dementia:
5. Reviews, meta-analyses and pooled analyses were not considered but could only have been used to find the proper original studies.

**Fig. 1:**
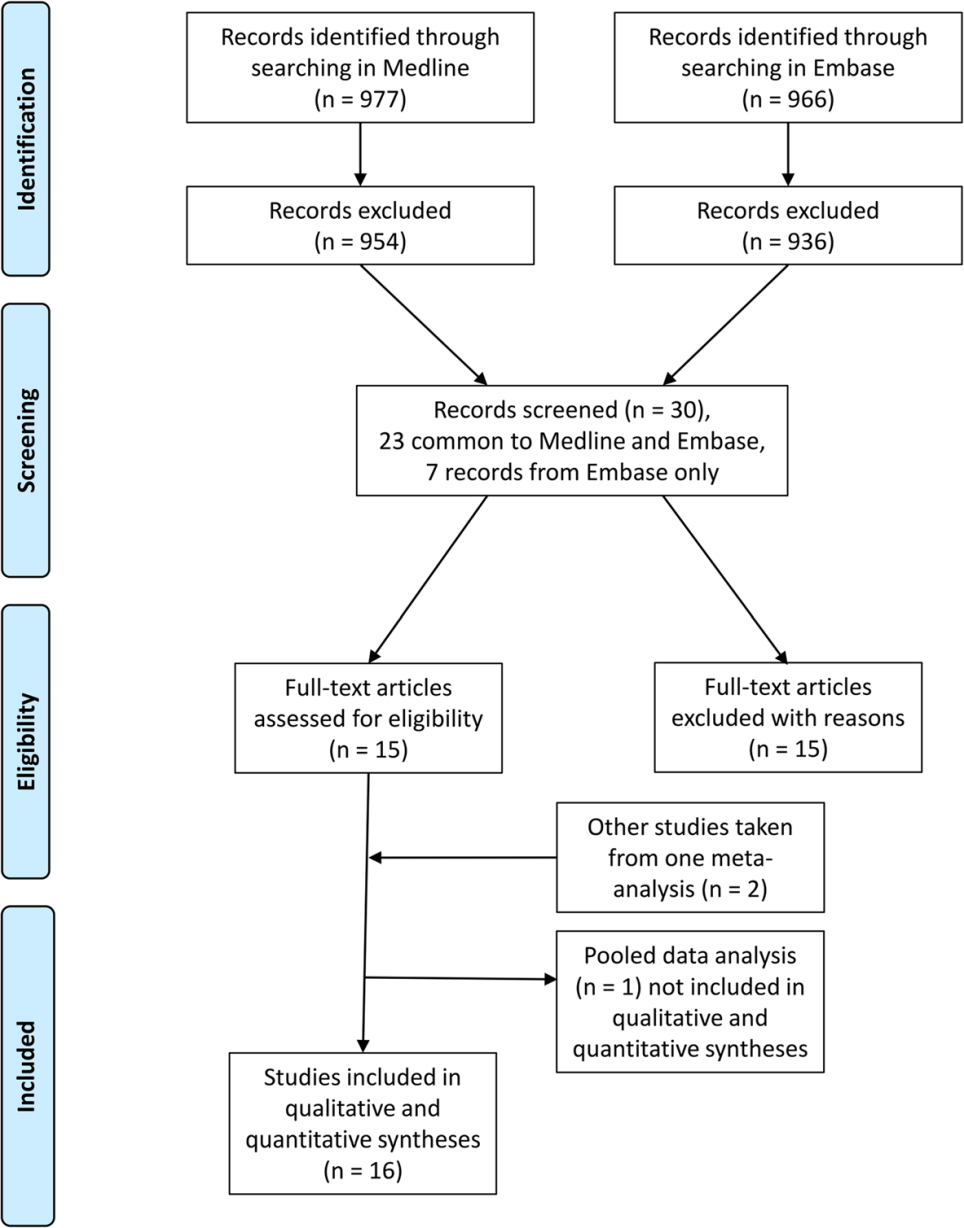
Overview of article selection (PRISMA 2009)

Two authors read the abstracts of all publications identified on the electronic databases, excluding only those that clearly did not meet the aforementioned eligibility criteria. In the next stage, four authors read the full-text versions of the selected publications and a consensus was reached regarding those remaining studies which met all criteria. The process of article selection followed PRISMA guidelines [8] and is illustrated in Fig. 1.

For evaluating the quality of the studies, we adopted the same approach described in the review by Prince et al. [4]. The scoring system used to assess the quality of the studies (which considered sample size, design, response proportion and diagnostic assessment; range 0–11) is presented in Appendix. In order to quantify the studies that adopted the DSM-IV and the National Institute of Neurological and Communicative Disorders and Stroke and the Alzheimer’s Disease and Related Disorders Association (NINCDS-ADRDA) criteria, the type of diagnostic tool and clinical criteria for the diagnosis of dementia were also considered [6]. When it was not clear how to classify the quality of some publications, the four authors came to a consensus following discussion. Zero points were assigned to items for which the scoring was not applicable due to lack of information. In order to globally evaluate the quality of the epidemiological data available regarding the prevalence of dementia and AD in Italy, we first scored each study and then calculated the mean, range of scores, standard deviation and median of the overall quality score.

## Results

Our systematic search yielded titles for 977 publications in Medline and for 966 publications in Embase. After reading the abstracts, 954 publications from Medline and 936 from Embase were excluded as clearly ineligible, leaving 30 articles for further review. Of these 30 articles, 23 were common to both libraries and seven were retrieved from Embase only. After obtaining copies of the fully published versions of each study, 15 publications were excluded (including all seven additional references from Embase) because they were deemed ineligible or were duplicates of publications already included.

We therefore analyzed 15 publications [9–23], comprising 14 different single studies and a pooled data analysis of 4 studies [23]. Two out of the four studies [10, 18] considered in the pooled data analysis [23] were already included in the list of the 14 single studies. Further to this, two other studies [24, 25] mentioned in the pooled analysis were also added into our analysis. These two studies did not focus on the prevalence of dementia and were not identified using the keywords used for our literature search; however, they did report data of interest. For these last two studies we integrated the data of interest obtained from the pooled analysis in Francesconi et al. [23] with those in the original publications. In this way, 16 of the 17 retrieved studies, i.e. all studies except the pooled data analysis [23], were included for the final analysis of the quality score (Fig. 1). Features of each study together with their quality score are outlined in Table 1. Finally, as shown in Table 2, only the studies included in the most comprehensive and frequently cited European publications on the prevalence of dementia in Europe were considered suitable for the quality analysis [1, 4, 6, 26, 27]. Out of 16 selected studies, 75 % (12 of 16) were published before the year 2000. The mean gap between the year of survey and year of publication was 4.1 ± 1.9 years (median, 4 years).

**Table 1:** Characteristics, scoring and prevalence of dementia/AD in studies carried out in Italy

First author and year of publication	Year of survey	Area of investigation	Type of dementia(s)	Age (years)	Sample size Score	Design Score	Response proportion Score	Diagnostic assessment Score	Total score	Prevalence of dementia	Diagnostic criteria tools
*Rocca, 1990* [9]	1987	Appignano (Macerata)	D (AD+MID+MD)	>59	778 Score: 1	Two-phase design with negative screen Score: 1	96 % Score: 3	AMT + MMSE+ Blessed-Roth+CE+IN Score: 3	8	6.2 % (2.6 % AD)	NINCDS-ADRDA HIS
*ILSA, 1997* [10]	1992–1993	8 municipalities	Any type	65–84	5632/5462 (total/eligible) Score: 2	Two-phase design with negative screen Score: 1	84-64 %^a^ Score: 3	IN + CE (MMSE/ADL/IADL) Score: 3	9	7.2 % F 5.3 % M	DSM-III-R NINCDS-ADRDA ICD-10
*Prencipe, 1996* [11]	1992–1993	Aquila Province	D (AD+VaD+ODD)	>64	1147 Score: 1	Two-phase design with negative screen Score: 1	84.4 % Score: 3	MMSE/MSQ + CE + IN + disability assessment Score: 4	9	8.0 % (5.2 % AD)	NINCDS-ADRDA NINDS-AIREN HIS
*De Ronchi, 1998* [12]	1991	Granarolo (Ravenna)	AD + VaD + M D	≥61	557 (481 completers) Score: 1	Two-phase design with no negative Score: 0	86.4 % Score: 3	MMSE/GDS + CE + IN + ADL Score: 2	6	11.1 %	DSM III R
*Benedetti, 2002* [13]	1996	Buttapietra (Verona)	AD + VaD	>74	238 Score: 0.5	One-phase design Score: 2	93.3 % Score: 3	MMSE + CE + IN + ADL Score: 3	8.5	15.8 % (6.7 % AD)	HIS NINCDS-ADRDA DSM-III-R
*Ferini-Strambi, 1997* [14]	1991	Vescovato (Cremona)	AD + VaD + MD + SeD	>59	856 (673 responders) Score: 1	Two-phase design with no negative Score: 0	79 % Score: 2	AMT + CE Score: 2	5	9.8 % (5.2 % AD)	NINCDS-ADRDA NINDS-AIREN
*D’Alessandro, 1996* [15]	1992	Troina (Enna)	D (VaD)	>74	365 Score: 0.5	Two-phase design with negative screen Score: 1	95 % Score: 3	MMSE + CE + CDR Score: 3	7.5	21.9 %	DSM-III-R HIS
*Azzimondi, 1998* [16]	1992–1994	2 Sicilian Communities (data on S. Agata Militello)	D (VaD)	>74	408 Score: 1	Two-phase design with negative screen Score: 1	93 % Score: 3	MMSE + CE + CDR Score: 3	8	28.4 %	DSM-III-RHIS
*Cristina, 2001* [17]	1992–1993	Pavia Province	D	>65 (40 % 65–69 and all >70)	2442 Score: 1.5	Two-phase design with negative sample Score: 1	68 % Score: 2	MMSE + IN + CE Score: 3	7.5	11.8 %	DSM-III-R
*Tognoni, 2005* [18]	2000	Pisa Province (Vecchiano)	VaD + AD+ LBD + MCI	>65	2366 Score: 1.5	Two-phase design with indirect sample of negative screen	68 % Score: 2	MMSE/CDR/CAMDEX + C E+ IN + ADL Score: 3	7.5	6.2 % (4.2 % AD)	NINCDS-ADRDA HIS LBD MCADRC DSM-IV
*Lucca, 2011* [19]	2002–2010	Monzino (Varese)	D (AD)	≥80 (80–100)	2316 Score: 1.5	One-phase design Score: 2	88 % Registered Score: 3	MMSE/BIMC/CDR + CE + IN+ disability assessment Score: 4	10.5	32 %	DSM-IV
*Ravaglia,1999* [20]	1994–1995	Bologna + Ravenna provinces	AD + VaD	≥100	154 Score: 0.5	One-phase design Score: 2	65 % Score: 2	MMSE + CE + IN+ disability assessment Score: 3	7.5	61.9 % (48.9 % AD)	DSM-IV NINCDS-ADRDA ICD 10
*Spada, 2009* [21]	2005–2006	San Teodoro (Enna)	AD + VaD + Others	60–85	374 Score: 0.5	Two-phase design with no negative screen sample Score: 0	74.9 % Score: 2	MMSE + CE + IN+ disability assessment Score: 3	5.5	7.1 % (4.1 % AD)	DSM IV NINCDS-ADRDA NINDS-AIREN
*Ravaglia, 2002* [22]	1999–2000	Conselice (Ravenna)	AD + VaD	65–97	1353 Score: 1	Two-phase design with negative screen sample Score: 1	75 % Score: 2	MMSE + CE + IN+ disability Assessment Score: 3	7	5.9 % (3.0 % AD)	DSM-IV NINCDS-ADRDA NINDS-AIREN
*Ferrucci, 2000* [24]	1998	Greve in Chianti + Bagno a Ripoli (Florence)	D and AD	>65–90+	1260 Score: 1	Two-phase design with negative screen Score: 1	91.6 %^b^ Score: 3	MMSE + CE + IN+ disability assessment Score: 3	8	7.1 % (3.6 % AD)^d^	DSM-III-R NINCDS-ADRDA
*Di Bari, 1999* [25]	1995	Dicomano (Florence)	D and AD	>65–90+	864 Score: 1	Two-phase design with negative screen Score: 0	91.2 %^b^ Score: 3	MMSE^c^ + MODA + CE + BADL Score: 3	7	9.0 % (5.2 % AD)^d^	Unknown

*General*: *F* females, *M* males, *NA* not available

*Type of dementia and other diseases*: *AD* Alzheimer Disease, *D* Dementia, *LBD* Lewy Body Dementia, *MCI* Mild Cognitive Impairment, *MD* Mixed Dementia, *MID* Multi-Infarct Dementia, *ODD* Other Dementing Diseases, *SeD* Secondary Dementia, *VaD* Vascular Dementia

*Area of investigation*: *SAM* community of Sant’Agata Militello

*Diagnostic assessment score*: *ADL* Activities of Daily Living, *AMT* Abbreviated Mental Test, *BADL* Bristol Activities of Daily Living, *BIMC* Blessed Information Memory Concentration, *CAMDEX* Cambridge Mental Disorders of the Elderly Examination, *CDR* Clinical Dementia Rating, *CE* Clinical Examination, *GDS* Global Deterioration Scale, *IADL* Instrumental Activities of Daily Living, *IN* Interview, *MDS* Minimum Data Set, *MMSE* Mini-Mental State Examination, *MODA* Milan Overall Dementia Assessment, *MSQ* Mental Status Questionnaire

*Diagnostic criteria tools*: *DSM* Diagnostic and Statistical Manual of Mental Disorders, *HIS* Hachinski Ischemic Score, *ICD* International Classification of Diseases, *MCADRC* Mayo Clinic Alzheimer’s Disease Research Center, *NINCDS-ADRDA* National Institute of Neurological and Communicative Disorders and Stroke-Alzheimer’s Disease and Related Disorders Association, *NINDS-AIREN* National Institute of Neurological Disorders and Stroke-Association Internationale pour la Recherche et l’Enseignment en Neurosciences, *RPM* Raven Progressive Matrix

^a^Response rates for personal interview and clinical evaluation, respectively

^b^Calculated on those who were traceable

^c^MMSE and adjustment tests when score falls between 22 and 25

^d^Estimated from Table 2 in the pooled analysis [23]

**Table 2:** ADI quality score included in the meta-analysis on the prevalence of dementia in Europe (1980–2014)

First author and/or name of survey	Range of time considered	Italian studies included	ADI quality score	ADI quality score (mean ± SD; median) (mean ± SD; median)
Hofman, 1991, EURODEM [26]	1980–1990	Rocca et al., 1990 [9]	8	8
Lobo, 2000, EURODEM [27]	1990–2000	ILSA, 1997 [10]	9	9
Reynish, 2006, EUROCODE [1]	1990–2007	Prencipe et al., 1996 [11]	9	7.3 ± 1.5; 7.5
		Ferini-Strambi et al., 1997 [14]	5	
		Azzimondi et al., 1998 [16]	8	
		Ravaglia et al., 2002 [22]	7	
		Tognoni et al., 2005 [18]	7.5	
Galeotti, 2013, ALCOVE [6]	2007–2011	Lucca et al., 2011 [19]	10.5	10.5
Prince, 2013 [4]	1980–2009	Rocca et al., 1990 [9]	8	7.4 ± 1.1; 7.5
		D’Alessandro et al., 1996 [15]	7.5	
		Prencipe et al., 1996 [11]	9	
		Ferini-Strambi et al., 1997 [14]	5	
		Azzimondi et al., 1998 [16]	8	
		De Ronchi et al., 1998 [12]	6	
		^a^Di Bari et al., 1999 [25]	8	
		Ravaglia et al., 1999 [20]	7.5	
		^a^Ferrucci et al., 2000 [24]	8	
		Cristina et al., 2001 [17]	6.5	
		Ravaglia et al., 2002 [22]	7	
		Benedetti et al., 2002 [13]	8.5	
		Tognoni et al., 2005 [18]	7.5	

*ADI*, Alzheimer Disease International, *ALCOVE* Alzheimer Cooperative Valuation in Europe, *EuroCoDe* European Collaboration for Dementia Group, *ILSA* Italian Longitudinal Study on Aging

^a^For the calculation of the ADI quality score, the items reported in these publications have been integrated with those reported in the pooled data of Francesconi et al. [23]

**Fig. 2:**
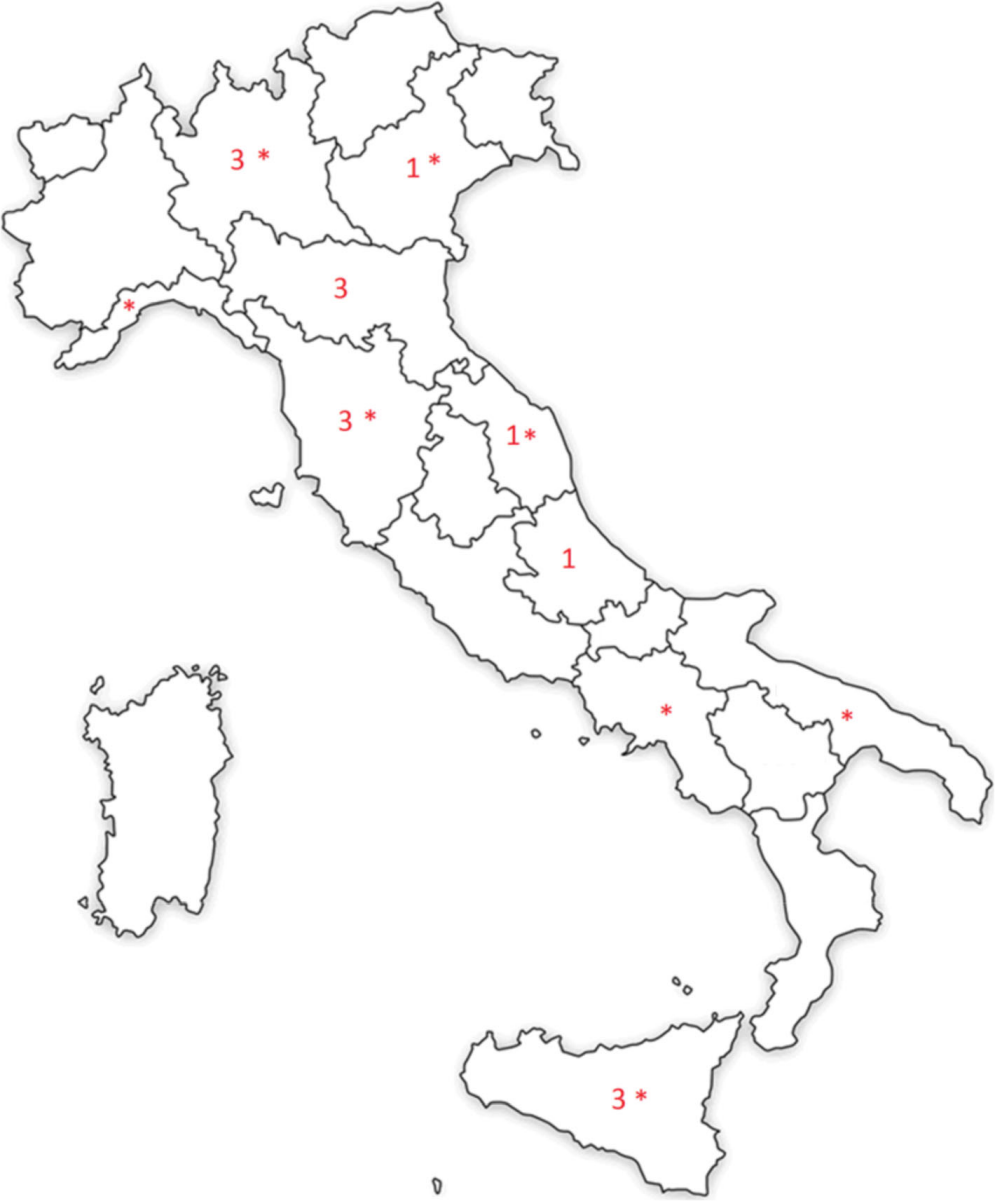
Geographic distribution and the relative number of Italian publications on prevalence of dementia (map created by authors). Note: The asterisks refer to the municipalities included in the ILSA study [10]

Apart from the Italian Longitudinal Study on Aging (ILSA) survey [10], most of the studies evaluated the prevalence of dementia in Northern Italy and Tuscany (10 of 16). Two studies were conducted in Central Italy [9, 11] and the remaining three studies were performed in Sicily [15, 16, 21] (Fig. 2). Even including the ILSA study, we did not find any data on the prevalence of dementia for 10 out of 20 Italian regions.

The prevalence of dementia in the Italian studies ranged from a minimum of 5.9 % (for a sample with range of 65–97 years) to a maximum of 61.9 % (for a sample with age >100 years) (Table 1). Out of the 16 studies included in this review, 13 reported prevalence by age and sex. Ten of 16 analyzed studies (62.5 %) reported the specific prevalence data for AD. We found in these 10 studies that the prevalence of AD increased with the age of the studied population (from 3 % for a range of age of 65–97 years old to 48.9 % for a study sample with age >100 years old) (Table 1). Only the ILSA study [10] had a sample size >3000 (Table 1). Twenty-five percent of the studies (4 of 16) considered 60 years to be the minimum age for inclusion (i.e. the conventional age threshold to define elderly), while the majority of studies used 65 years as the age threshold.

The majority of studies (62.5 %; 10 of 16) reported a response rate greater than or equal to 80 %. Regarding study design, only three studies (18 %) were performed with a one-phase design method [13, 19, 20]. Of the remaining studies, 13 had a two-phase design and 10 were conducted with sampling of screen negatives. No studies adopted the weighting back method (for all information see Table 1).

**Fig. 3:**
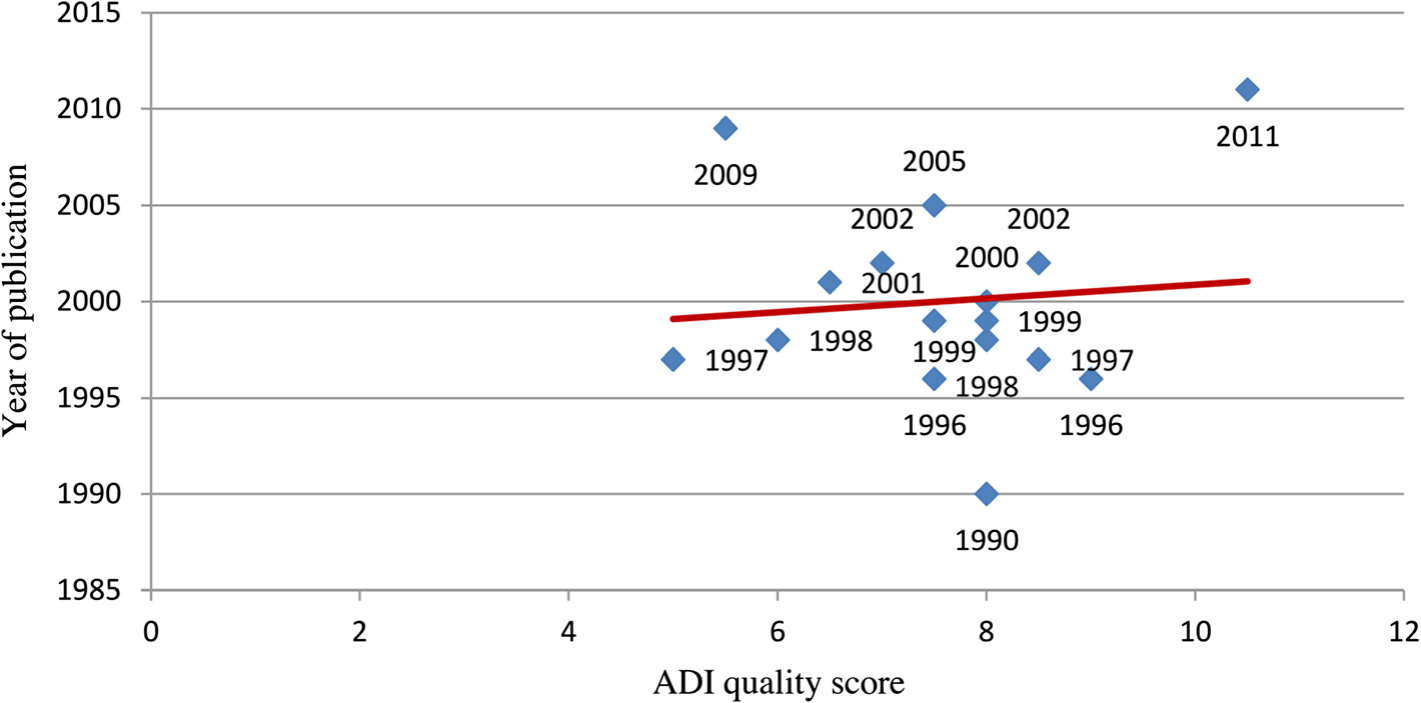
Relationship between the quality of studies on the prevalence of dementia in Italy and the year of publication. *ADI*, Alzheimer Disease International

Table 1 also indicates the diagnostic tools used in the 16 studies. With regard to diagnostic assessment, the informant interview was performed in three of the 16 studies. A total of six studies adopted the NINCDS-ADRDA criteria, four studies adopted both the NINCDS-ADRDA and the DSM-IV criteria, and one study was performed with DSM-IV alone. The assessment tool used was not reported in one of the remaining five studies, whilst in four studies neither the DSM-IV nor the NINCDS-ADRDA criteria were utilized. Overall, the 16 epidemiological studies scored a mean ADI quality score of 7.6 ± 1.4 with a median of 7.75. As shown in Fig. 3, there was only a slight tendency for study quality to improve over time. When only the studies included in the Prince et al. [4] analysis were considered, the mean quality score of the Italian studies was found to be numerically less than that of the European studies (7.4 ± 1.1 vs. 8.2 ± 1.8) (Table 2).

## Discussion

### Summary of evidence

Overall, this systematic review showed that the analyzed studies do not represent a valid source of epidemiological data on the prevalence of dementia in Italy. We found that, until now, no epidemiological data for 10 out of 20 Italian regions were available and that the majority of epidemiological studies were performed at the level of municipalities, with most studies conducted in Northern Italy. Some regions were more affected by dementia than others.

Irrespective of geographical distribution, the prevalence rates of dementia reported in the Italian studies vary widely which may be due to important differences in methodological approaches and population age ranges. In the 16 studies analyzed, we found five different age ranges of study samples, a discrepancy that makes it difficult to compare the results of these studies and suggests a lack of methodological consensus. Furthermore, it is important to note that the only nationwide survey on the prevalence of dementia in Italy with a sample size >3000 subjects excluded those who were older than 84 years, an age range associated with a high rate of dementia.

From a diagnostic methodological perspective, the majority of Italian studies on the prevalence of dementia and AD included in our analysis adopted a two-phase design, but not all of them used sampling of screen negatives and none of them adopted the weighting back method. Furthermore, the informant interview was performed in only a minority of selected publications.

Overall, the Italian studies included in this review had lower ADI quality scores than those of European studies and, unlike the finding reported in the meta-analysis of Prince et al. [4], quality showed only a slight tendency to improve over time.

#### Implications

The finding of the lack of robust recent epidemiological data is in accordance with the global data reported in a meta-analysis, which showed that the number of epidemiological studies on the prevalence of dementia in high-income countries peaked in the 1990s and subsequently dropped off sharply [4]. Even if the prevalence of dementia and AD has not changed significantly over time [1], the paucity of epidemiological data on the prevalence of dementia in Italy over the last ten years is regrettable and has important implications from economic and social points of view. Indeed, annual updates of the actual number of patients with dementia residing in a country should be the first step in creating a policy supporting patients and their families. It is also noteworthy that the geographic distribution of territorial Alzheimer Evaluation Units in Italy is not homogeneous, with the majority located in the north of the country [28]. This geographic distribution might explain, at least in part, why most epidemiological studies on dementia and AD in Italy have been performed in northern regions.

Another issue regarding studies on the prevalence of age-related diseases like dementia and AD is the timing of publication in relation to the time of survey. At a national level, health policy strategy is dependent on accurate and current estimates of the size of the problem [1, 4]. The gap between the dates of the surveys and their dates of publication, together with the scarcity of recent data, suggests that the available publications on the prevalence of dementia in Italy may not represent an up-to-date source of information for health economic policy planning regarding patients with dementia.

Epidemiological studies on prevalence of dementia in Italy show low methodological quality. As Prince et al. [4] reported, multiphase methods in general tend to underestimate the prevalence of dementia and overestimate the precision. In accordance with other epidemiological studies [4], our analysis confirmed that many studies omitted the informant interview. Furthermore, prevalence estimates may reflect the diagnostic criteria adopted by each study. For example, a study that evaluated the prevalence of dementia using different systems of classification found that the proportion of subjects with dementia varied from 3 % when International Classification of Diseases (ICD)-10 criteria were used to 29 % when DSM-III criteria were applied [7]. Similarly, the variability observed in European epidemiological studies has been attributed precisely to the clinical criteria adopted [1, 6]. In our analysis, 31 % of the studies used neither the DSM-IV criteria nor the NINCDS-ADRDA criteria. This finding represents a major methodological issue considering that only the latter diagnostic criteria have been validated with post-mortem data [6].

The weak ADI quality scores of the Italian studies, along with evidence that quality showed only a slight tendency to improve over time, has important implications at the national healthcare system level. Since no national survey commissioned by the Italian government has been performed in Italy, we suggest that the Italian healthcare system should urgently institute nationally representative surveys using the highest quality epidemiological methods, as defined in the ADI 2009 report, and repeat them at regular intervals to track any changes in the prevalence of dementia or AD [4, 6].

Based on the findings of our systematic review, we believe that the development of a national plan might be an appropriate strategy to obtain epidemiological estimates on dementia using the current healthcare system and, at the same time, we encourage researchers to undertake national surveys. A national plan might help overcome differences between Italian regions, whilst the detailed estimates obtained in this way might be useful for policymaking, planning, and allocation of health and welfare resources.

#### Limitations

This review has several limitations First, our selected studies included surveys that were not specifically dedicated to the prevalence of dementia [10, 24, 25], which may have resulted in a bias in the types of publications included in the review. Second, although we reported that 75 % of studies were published before the year 2000, this finding might be due to our search methodology as PubMed/Medline and Embase were the only databases searched. However, this bias is unlikely to be substantial since all studies included in our analysis (Table 2) were also included in the most relevant meta-analysis published in this field [4] Third, the quality of studies included in this review was low. Fourth, the mean gap of four years between the year of survey and the year of publication should also be taken into account. Fifth, the review was not listed on an international prospective register of systematic reviews such as PROSPERO [29]. Sixth, this review has the intrinsic methodological limitation that the prevalence rates derived from all the analyzed studies have not been standardized or compared with those of a reference population, e.g. one chosen for age and sex. Finally, it should also been taken into consideration that although the quality of the studies only slightly improved over time, our literature search for the studies on prevalence began in 1980 and 75 % of the selected studies were published prior to 2000. Therefore, many of the included studies were unlikely to have been conducted in conformity with current requirements for epidemiological studies [30].

## Conclusions

Despite the availability of several publications, data on the prevalence of dementia in Italy and their usefulness for evaluating the epidemiological burden of the disease in Italy are minimal. The majority of studies were conducted in the 1990s with important methodological and geographic differences that undermine determination of the true national prevalence of dementia. Overall, the quality of Italian studies was lower than that of European studies and only slightly improved over time. Full implementation of a Dementia National Plan would help physicians, scientists and regulators to better understand the epidemiology of dementia and AD in Italy.

## Appendix

**Table 3:** Standardized scoring system for the assessment of quality of epidemiological trials in dementia [5]

Item	Score
Sample size	
<500	0.5 points
500–1499	1 point
1500–2999	1.5 points
≥3000	2 points
Design	
Two-phase study with no sampling of screen negatives^a^	0 points
Two-phase study with sampling of screen negatives but no weighting back	1 point
One-phase study or two-phase study with appropriate sampling and weighting	2 points
Response proportion	
<60 %	1 point
60–79 %	2 points
≥80 %	3 points
Diagnostic assessment	
Inclusion of multidomain cognitive test battery, formal disability assessment, informant interview and clinical interview	1 point each

^a^In the two-phase study, all participants are evaluated in the first phase using a screening tool. All the patients with a score below a predefined cutpoint (screen positives) will enter into the second phase of the study for a more comprehensive evaluation. In order to get a more correct evaluation, a random sample with a score above the cutpoint (screen negatives) should also be included in the second phase of the study. In this way the false positive rate can be estimated among the screen negatives and the related weight (‘weight back’) can be evaluated, calculating an overall prevalence taking into account the different sampling proportions of screen positives and screen negatives. In the one-phase study, all patients directly receive a comprehensive clinical evaluation

## Abbreviations

AD: Alzheimer Disease
ADI: Alzheimer Disease International
ALCOVE: Alzheimer Cooperative Valuation in Europe
DSM: Diagnostic and Statistical Manual of Mental Disorders
EuroCoDe: European Collaboration for Dementia Group
ICD: International Classification of Diseases
ILSA: Italian Longitudinal Study on Aging
NINCDS-ADRDA: National Institute of Neurological and Communicative Disorders and Stroke-Alzheimer’s Disease and Related Disorders Association
